# Bird nesting boxes as a specific artificial microenvironment increasing biodiversity of mites from the suborder Uropodina (Acari: Mesostigmata): a case study of Bory Tucholskie National Park

**DOI:** 10.1007/s10493-024-00912-9

**Published:** 2024-06-13

**Authors:** Jerzy Błoszyk, Jacek Wendzonka, Marta Kulczak, Karolina Lubińska, Agnieszka Napierała

**Affiliations:** 1 Department of General Zoology, Faculty of Biology, Adam Mickiewicz University, Uniwersytetu Poznańskiego 6, 61-614 Poznań, Poland; 2 Natural History Collections, Faculty of Biology, Adam Mickiewicz University, Uniwersytetu Poznańskiego 6, 61-614 Poznań, Poland; 3 Bory Tucholskie National Park, 89-606 Charzykowy, Poland

**Keywords:** Community structure, Microhabitat, Nest of birds, Nidicole, Protected area

## Abstract

Bory Tucholskie National Park, founded in 1996, is one of the most recently established national parks in Poland, and therefore, has not been thoroughly examined yet. The authors of the current study present results of their research concerning communities of mites from the suborder Uropodina (Acari: Mesostigmata), inhabiting bird nesting boxes within the area of Bory Tucholskie National Park. The mite community comprises two nidicolous species, i.e. *Leiodinychus orbicularis* (C.L. Koch, 1839) and *Chiropturopoda nidiphila* (Wiśniewski and Hirschmann 1993). The former is a species characteristic of various types of nests, as well as nesting boxes, where it is usually the eudominant species. The latter is an extremely rare and scarce species of Uropodina, known thus far from woodpeckers’ hollows. The population of *L. orbicularis* in the analysed communities in the realm of Bory Tucholskie National Park has been estimated to be over 6,000 specimens, and in the case of *Ch. nidiphila -* over 400 specimens.

## Introduction

A nest box (birdhouse) is a specially prepared construction, usually made of wood and intended for nests, mainly for birds (Aitken and Martin 2007; Cockle et al. 2012; Walankiewicz et al. 2014; Zawadzka 2018), but also for bats (Rueegger 2016), dormice (Gliridae) (Morris et al. 1990; Koppmann-Rumpf et al. 2003), and hymenoptera (MacIvor 2017), which do not have enough suitable nesting places in the environment transformed by humans. Due to the shortage of adequate nesting places, often it happens that animals occupy nest boxes originally intended for other species, as is the case of dormice, which sometimes inhabit birdhouses (Błoszyk et al. 2023). Nest boxes serve not only rare vertebrates, for which they are usually built, but also promote growth in biodiversity of other animal groups, especially invertebrates. It has been shown on multiple instances that birdhouses inhabited by birds are also willingly inhabited by representatives of other systematic groups, including Arachnida, Isopoda, Gastropoda, Myriapoda, and numerous Insecta (especially species of Coleoptera, Diptera, Siphonaptera, Hemiptera, Hymenoptera and Lepidoptera), (Nordberg 1936; Woodroffe 1953; McComb and Noble 1982; Tajovský et al. 2001; Turienzo et al. 2010; Krištofík et al. 2013; Broughton et al. 2015; Boyes 2018; Boyes and Lewis 2018; Jaworski et al. 2022). One of the most numerous groups of arthropods found in this type of microhabitat are mites. Some of them, like Acaridae (Solarz et al. 1999), are very abundant in birdhouses. Furthermore, species from the order Mesostigmata are highly populous in birdhouses, among which are ectoparasites (Bajerlein et al. 2006; Gwiazdowicz and Matysiak 2004; Mašán et al. 2014; Błoszyk et al. 2016). However, obligate ectoparasites, such as ticks, are rarely found in bird houses (Gwiazdowicz and Matysiak 2004). Among the Mesostigmata found in this microhabitat are also species from the suborder Uropodina (Acari: Mesostigmata). They have already been the object of research focused on birdhouses of various bird species and dormice (Błoszyk and Olszanowski 1985, 1986; Kaczmarek and Pajkert 1987; Mazgajski 2007; Napierała and Błoszyk 2013; Błoszyk et al. 2016, 2020, 2023), however each subsequent observation based on a large amount of material reveals new, previously unknown information about the species composition and structure of Uropodina communities inhabiting such microhabitats.

The research presented in this study was conducted within the area of Bory Tucholskie National Park (BTNP) in Northern Poland, which was established in 1996, and is one of the newest national parks in Poland (Park Narodowy Bory Tucholskie 2014). The park is also one of the less effectively examined protected areas in terms of acarofauna. None of the studies summarizing the state of research on Uropodina in national parks in Poland (Błoszyk 1991; Wiśniewski 1996) takes this location into account. Moreover, in the publications from 2004 to 2006 presenting the state of research on the acarofauna of Ojcowski National Park in comparison to other national parks in Poland (Błoszyk et al. 2004; Napierała et al. 2006), BTNP was not included due to the lack of data on mites from this area. The first remarks concerning mites from the suborder Uropodina found in this location can be found in the work by Gwiazdowicz and Matysiak (2004), where they list 10 species of Uropodina from selected microhabitats in this park, found mainly in dead wood.

The major aim of the study presented here was to ascertain the structure of Uropodina communities inhabiting nest boxes in the area of BTNP and to assess to what extent their presence affects the overall biodiversity of this group of mites in the examined area. While cleaning the boxes and collecting the material for the study, in some the presence of bat guano was recorded, indicating that they were visited by bats. Therefore, the next objective of the study was to assess how the presence of bat guano affects the structure of Uropodina communities in bat-visited and non-visited boxes. Thus, having at their disposal material from nest boxes (collected for the first time for acarological research purposes in the area of BTNP), the authors were able to supplement the information concerning the acarofauna of this poorly examined national park.

## Materials and methods

### Study area

The material for the study was collected in the area of BTNP (53°49’N 17°34’E), which encompasses an area of 4,613.04 ha. Most of this area (83%) is covered by forest ecosystems. Therefore, the area of the park, with the buffer zone, constitutes one of the largest, dense forest complexes in Poland (Dz.U. z 1996 r. Nr 64, poz. 305). The largest area of the park is under partial legal protection, i.e. 4,209.78 ha (91%). The legal protection is valid in areas where the main objects of preservation require active protection. The remaining area of the park, 324.30 ha (7% of the whole area), has been under strict protection. This area is defined by the greatest stability and resistance to degradation, and is also under landscape protection (78.96 ha), which constitutes 2% of the whole protected area (Dz.U. z 2008 r. Nr 230, poz. 1545). The exceptional natural value of this area stems from the presence of a unique landscape both in Poland and Europe– the outwash lake district (sand and lake district). The most valuable elements of the BTNP are the lobelia lakes, as well as the peatbogs and pine forests. In total, there are 24 lakes with a total area of 5.37 km^2^ (Sojka et al. 2021). In addition, a few zones have been delimited as to protect refuges, sites of plants, lichens and fungi under species protection, as well as breeding places and sites with regular residence of legally protected animals. Among the plants, *Luronium natans* occurring (L.) Raf.in the lakes in the area of the park is protected. There are also valuable lichens present such as *Usnea subfloridana* Stirt., *Usnea hirta* (L.) Weber ex F.H. Wigg., and *Usnea filipendula* Stirt. In the vicinity of the park there are zones intended for the protection of refuges, breeding sites, as well as the regular residence of the white-tailed eagle (*Haliaeetus albicilla* L.) and eagle owl (*Bubo bubo* L.) (Dz.U. z 2008 r. Nr 230, poz. 1545).

All nest boxes from which material for the study was obtained were 25.5 × 20.5 × 26.5 cm (type A1). The boxes were placed at a height of 2.5–3.5 m in a similar forest ecosystem– a young moderate forest. In this case, young tree stands were chosen because they often lack natural hollows that birds can use to establish their nests. The age of the trees in the divisions, where the boxes were hung in 2023, ranged from 52 to 81 years (the average age of the trees was between 60 and 70 years). The dominant species in all divisions was pine (*Pinus sylvestris* L.), with tree closure ranging from moderate to full. The bonitation was variable, usually 20 (II), 30 (III), and 35 (III.5). The undergrowth was dominated by a moss layer, bilberry plants (*Vaccinium myrtillus* L.), reindeer moss, and grasses.

### Data collection

**Fig. 1:**
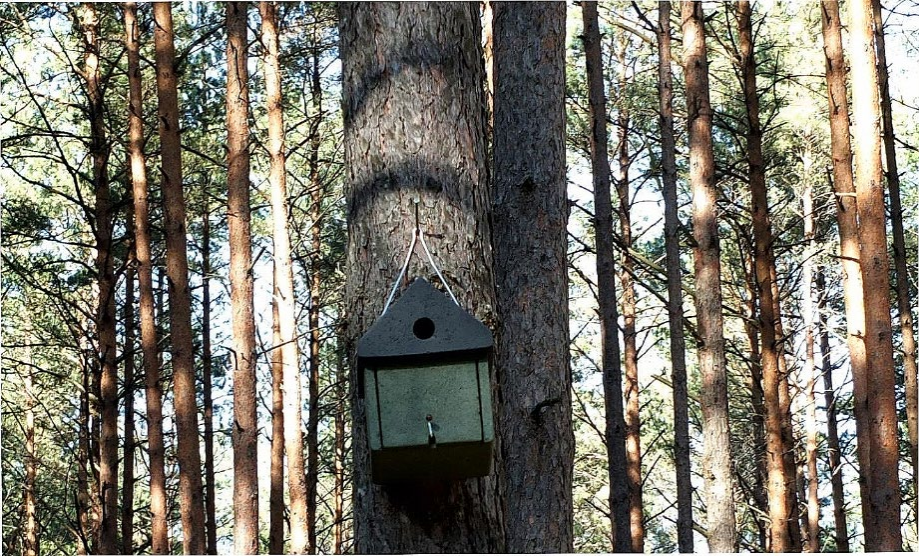
A sawdust-concrete nest box (type A1) hung in the area of Bory Tucholskie National Park (phot. by K. Lubińska)

**Fig. 2:**
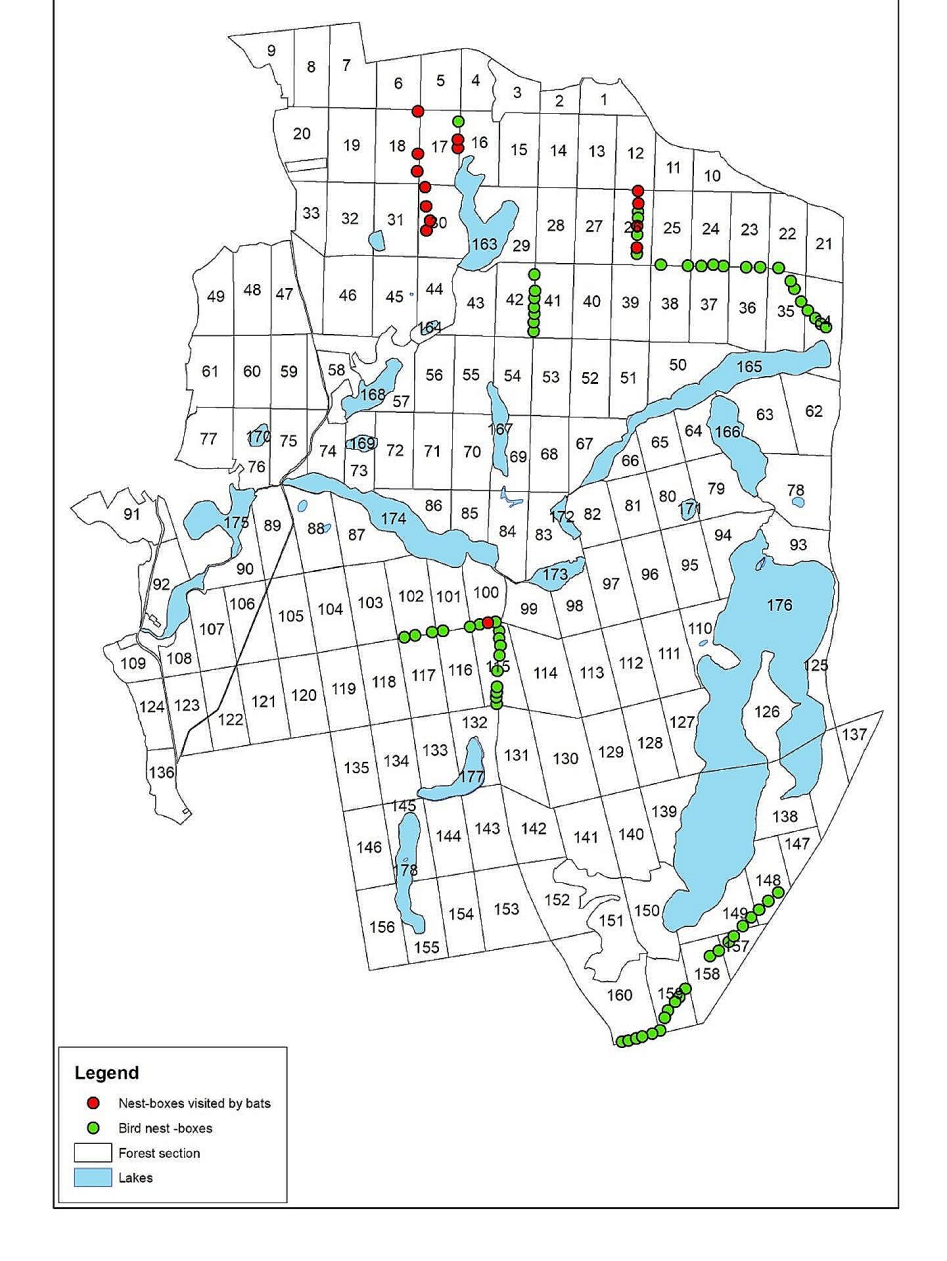
Distribution of examined nest boxes in the area of Bory Tucholskie National Park

The research material for the current study comes from 77 sawdust-concrete nest boxes (Fig. 1) hung in 2018 in the area of BTNP (Fig. 2). The research material was obtained on October 17–18, 2023, as part of the annual birdhouse cleaning campaign.

The nest material obtained from the examined nest boxes was placed in plastic zipper bags and transported from the park headquarters (Charzykowy) to Poznań, where the extraction of specimens was conducted for five days by use of Berlese-Tullgren funnels in artificial light (40 watts). Mites were sorted and identified with a stereoscopic microscope Olympus SZX 16. Open slides (Grandjean technique) have been made for juvenile stages and identified with a microscope Olympus BX53 with Nomarski Contrast. Identification of the extracted mites was conducted by the first author based on publications by Karg (1989), Błoszyk (1999), and Mašán (2001). Extracted specimens were then stored in Eppendorf tubes filled with c. 75% ethanol. The preserved samples have been stored in the Natural History Collections (Faculty of Biology) at Adam Mickiewicz University (AMU) in Poznań.

### Data analysis methods

The structure of the analyzed mite communities found in the examined nest boxes was characterized with the index of dominance (D), and frequency of occurrence (F). The scale has the following classes: dominance D5 eudominants (> 30.0%), D4 dominants (15.1–30.0%), D3 subdominants (7.1–15.0%), D2 recedents (3.0–7.0%) and D1 subrecedents (< 3.0%); frequency F5 euconstants (> 50.0%), F4 constants (30.1–50.0%), F3 subconstants (15.1–30.0%), F2 accessory species (5.0–15.0%) and F1 accidents (< 5.0%) (Błoszyk 1999). The data have been stored in the computer database Analizator 2.0 in the Natural History Collections (Faculty of Biology) at AMU.

The Maturity Index (MI) is a sensitive bioindication tool for monitoring forest ecosystems. It is based not only on the, traditional measurements, such as the concentration of heavy metals in soil and litter, but on the occurrence of species with *K* and *r*-selected life histories (Ruf 1998; N’Dri et al. 2018). In our study, the system is based on classifying mites from the suborder Uropodina according to their life-history traits on the *r*/*K* scale, with values ranging from 1 to 3. The assignment of particular Uropodina species was carried out according to such features as the ecological indices (dominance and frequency), ecological tolerance based on habitat, population growth rate, occurrence of larvae, and the presence and intensity of phoresy (Napierała and Błoszyk 2021).

The MI for communities of Uropodina was calculated as the weighted proportion of the *K*-selected species in the entire Uropodina community. The value of the index should be higher in less environmentally disturbed areas. The minimum value of the MI is zero (no *K* strategists at the site), and the maximum value is 1 (then all species are *K*-strategists).The MI of the BTNP was calculated based on the formula (Ruf 1998):

$$ MI=\frac{\sum _{i=1}^{S}Ki}{\sum _{i=1}^{S}Ki+\sum _{i=1}^{S}ri}$$

with S– number of species, K– K-value ranging from 1 to 3, r– r-value ranging from 1 to 3 for the species i. The differences between the average numbers of Uropodina in boxes inhabited exclusively by birds, as well as boxes visited by bats were tested with the non-parametric Mann-Whitney U Rank Test. The map in the Fig. 2 is original and generated with Corel Draw 2020 computer graphics software.

## Results

The Uropodina communities found in the nest boxes examined in the area of BTNP consisted of only two species of Uropodina, i.e. *Leiodinychus orbicularis* (C.L. Koch, 1839) and *Chiropturopoda nidiphila* (Wiśniewski and Hirschmann 1993), with a clear dominance of the former (see Table 1). The obtained results suggest that *L. orbicularis* was quite common (F = 44%) and very numerous (D = 99%) in the examined nest boxes. In the analysed samples, the presence of 1 to 1,028 specimens of this species was recorded (with an average of 57 specimens per sample). In this community, a clear predominance of males of this species has been observed (sex ratio 1:1.5), and juvenile forms are more than twice as numerous as adult forms.

**Table 1:** Community structure of Uropodina mites in bird nest boxes examined in the area of Bory Tucholskie National Park: N– number of specimens, F– females, M– males, D– deutonymphs, P– protonymphs, L– larvae, D% - dominance, F% - frequency, Ave ± SD– average ± standard deviation

Species	N	F	M	D	P	L	D%	F%	Ave±SD
*Leiodinychus orbicularis* (C.L. Koch, 1839)	1924	232	352	676	594	70	99.18	44.16	56.59±183.82
*Chiropturopoda nidiphila* (Wiśniewski & Hirschmann, 1983)	16	3	5	8	-	-	0.82	7.79	2.67±2.66
Total	1940	235	357	684	594	70			

*Chiropturopoda nidiphila* occurred in the examined nest boxes less frequently and less numerously than the other species. In the analysed material, one sample contained from 1 to a maximum of 8 specimens (with the average of 2.7 individuals per sample). Moreover, in the case of this species, a predominance of males has been observed in the analysed material (sex ratio 1:1.7). The only juvenile form found in the samples was deutonymphs, with a 1:1 ratio to the adult forms.

### Habitat preferences of *L. orbicularis* in bat-visited nest boxes

The results of the current study show that the boxes visited by bats were more frequently and numerously inhabited by *L. orbicularis* (see Table 2). The average number of Uropodina in the examined nest boxes visited and those not visited by bats was considerably different in each case: Mann-Whitney U Rank Test U = 244, z = 2.6 *p* < 0.01. This difference likely resulted from the number of *L. orbicularis*, which also differed very clearly between nest boxes visited and those not visited by bats. The difference was statistically significant, i.e. the Mann-Whitney U Rank Test U = 247, z = 2.66; *p* < 0.05. No significant differences were observed in the number of *Ch. nidipila* in the boxes visited and those not visited by bats, i.e. the Mann-Whitney U Rank Test U = 332, z = 1.44; *p* > 0.05.

**Table 2:** NS-number of samples, N-number of specimens, D%-dominance, F%-frequency, Ave ± SD-average ± standard deviation, Max-maximum number of specimens in a sample

Species	Nest boxes not visited by bats					
	NS	N	D%	F%	Ave±SD	Max
*L. orbicularis*	63	268	96.4	38.1	11.2±15.2	50
*Ch. nidiphila*	63	10	3.6	3.2	5.0±4.4	8
Total	63	278	100.0	38.0	4.4±11.4	
	**Nest boxes visited by bats**					
*L. orbicularis*	14	1,656	99.6	71.4	165.6±323.4	1,028
*Ch. nidiphila*	14	6	0.4	28.5	1.5±0.6	2
Total	14	1,662	100.0	71.4	118.7±280.6	

### Importance of nest boxes for Uropodina biodiversity in Bory Tucholskie National Park

Based on previous (Gwiazdowicz and Matysiak 2004) and current studies in BTNP, 19 species from the suborder Uropodina have been found to date (see Table 3). Two species reported in the current study, found in the examined nest boxes, were not mentioned in earlier studies (Gwiazdowicz and Matysiak 2004). This means that the presence of nest boxes as a new, in this case human-made microhabitat, contributes to the overall biodiversity of these mites in this location. This was also confirmed by the assessment of the park conservation value carried out based on the MI (Napierała and Błoszyk 2021). The value of this index, which is based on the life strategies of mites, for all species found in BTNP was 0.32, and is lower (MI = 0.28) when the calculation does not take into account the species found in the examined nest boxes.

**Table 3:** List of Uropodina species recorded to date in Bory Tycholskie National Park based on earlier studies and our own research (including their life-history strategies)

Species	Source	Strategy*	Value
*Trachytes aegrota* (C.L. Koch, 1841)		r1	1
*Oodinychus ovalis* (C L. Koch, 1839)		r1	1
*Pulchellaobovella pulchella* (Berlese, 1904)		r2	2
*Pulchellaobovella pyriformis* (Berlese, 1920)	Gwiazdowicz and Matysiak 2004	r3	3
*Urodiaspis tecta* (Kramer, 1876)		r1	1
*Olodiscus minima* (Kramer, 1882)		r1	1
*Olodiscus misella* (Berlese, 1916)		r2	2
*Oodinychus karawaiewi* (Berlese, 1903 )		r1	1
*Trachytes pauperior* (Berlese, 1914)		r1	1
*Dinychus arcuatus* (Trägårdh, 1922)		r3	3
*Polyaspis patavinus* Berlese, 1881		K1	1
*Polyaspis sansonei* Berlese, 1916		K1	1
*Trachyuropoda coccinea* (Michael, 1891)		K2	2
*Dinychus woelkiei* Hirschmann et Zirngiebl-Nicol, 1969		K1	1
*Dinychus carinatus* Berlese, 1903	Gwiazdowicz and Matysiak 2004	r2	2
*Trichyuropoda longiovalis* (Hirschmann and Zirngiebl-Nicol, 1961)	Gwiazdowicz and Matysiak 2004	K1	1
*Trichouropoda rafalskii* Wiśniewski and Hirschmann, 1984	Gwiazdowicz and Matysiak 2004	K1	1
*Leiodinychus orbicularis* (C.L. Koch, 1839)		r3	3
*Chiropturopoda nidiphila* (Wiśniewski and Hirschmann, 1983)		K3	3
Sum of *K*-species		10	
Sum of *r*-species		21	
Sum of r-sp.and K.sp		31	

* based on: Napierała and Błoszyk 2021

## Discussion

The Uropodina community in the examined nest boxes in the area of BTNP consists of two species of Uropodina mites, i.e. *L. orbicularis* and *Ch. nidiphila*. Earlier studies have shown that *L. orbicularis* is a granary mite and does not parasitise birds (Radinovsky 1965). It is present in farmyard manure, decaying leaves, the top layer of soil, mosses, as well as in cereals, anthills, and nests of moles and birds (Karg 1989; Wiśniewski and Hirschmann 1993; Mašán 2001; Napierała and Błoszyk 2013; Błoszyk et al. 2016). This species is also a typical nidicole inhabiting various types of nest boxes. It was also recorded in nest boxes of birds and bats (Błoszyk and Olszanowski 1985, 1986; Błoszyk et al. 2005, 2020; Napierała and Błoszyk 2013). In nest boxes, it is usually eudominant. The highest dominance of this species has been observed in bat boxes (F = 99%); nest boxes occupied by dormice (*Glis glis* (L.) and *Muscardinus avellanarius* (L.) (F = 99%); nests of tits (Paridae sp.) and flycatchers (*Muscicapa* sp.) in boxes (F = 98%); nests of starlings (Sturnidae sp.) in boxes (F = 84%) (Błoszyk et al. 2023). Similarly, in the case of nest boxes hung in BTNP, specimens of this species also constitute 99% of the whole Uropodina community. The frequency of *L. orbicularis* within the range of 44%, that has been observed in nest boxes in BTNP, is one of the highest ever recorded in Poland. It is similar to the frequency (F = 46%) of this species in nest boxes occupied by dormice (*G. glis* and *M. avellanarius*) and nests of the white stork (*Ciconia ciconia* (L.) (F = 32%) (Błoszyk et al. 2023).

In the analysed material, all developmental stages of *L. orbicularis* were found (Table 1). This result confirms previous observations on communities of Uropodina from boxes occupied by dormice, where all developmental stages of *L. orbicularis* (except for larvae) were also found (Błoszyk et al. 2023). This indicates that *L. orbicularis* undergoes a full life cycle in nest boxes. Deutonymphs of this mite species enter the boxes through phoresy carried out by insects. As in the case of *Uropoda orbicularis* (Müller 1776), a phenomenon of deutonymph accumulation is also observed here, which involves decreasing the pace of the deutonymphs’ transition into the adult form, as to increase the chances of successful species dispersion (Bajerlein and Błoszyk 2004).

The second species, *Ch. nidiphila*, was described by Wiśniewski and Hirschmann in 1993 based on only one deutonymph found in Wielkopolska (Greater Poland) (see Wiśniewski and Hirschmann 1993). The species is associated with tree hollows excavated by woodpeckers, which was proved in later research in Lower Silesia (Błoszyk et al. 2021), indicating that it is also a typical nidicole. The examined site in BTNP is the third record of this species in Poland (Wiśniewski and Hirschmann 1993; Błoszyk et al. 2021), and at the same time this location is the northernmost place of occurrence of this species in the country. The species occurs very rarely and in low abundance in the surveyed nest boxes in BTNP, and it is still unknown how it enters these microhabitats. The morphology of the anal region of the *Ch. nidiphila* deutonymphs does not indicate the possibility of producing a pedicel, and thus, phoresy on insects seems impossible in this case. That is why it is most likely that these mites are carried in the feathers of birds or in the fur of bats. However, due to the rare occurrence of these mites, they have never been found on their carriers. It is worth noting that all known species of this genus are related to bats in some way (Wiśniewski and Hirschmann 1993), but the essence of this relationship has not been elucidated. Both in the case of previously studied woodpecker hollows (Błoszyk et al. 2021), as well as the material from nest boxes discussed in this study, *Ch. nidiphila* is found in habitats visited by bats, which also left their guano there. Moreover, in the case of *L. orbicularis*, which was dominant in the surveyed boxes, its abundance was significantly higher in boxes visited by bats (Table 2). These boxes were located mainly in the northern part of the park, usually near wider areas without trees (Fig. 2), which are most likely the flight routes of bats during their hunting for insects at night.

According to the evaluation of the threat level of individual Uropodina species in Poland published by Napierała et al. (2018), the status of *L. orbicularis* was defined as Near Threatened (NT). However, both the study presented here, as well as a more recent study published by Błoszyk et al. (2023), show that this species is very abundant and frequent in various types of nests and nest boxes inhabited by various animals. Considering the number of nests naturally established by birds, as well as the number of nest boxes made by humans, this may be enough to provide this species with numerous suitable microhabitats. Thus, the conservation status of this species is presumably better and perhaps should be assigned to the Least Concern (LC) category. However, this requires further research and observations. Regarding *Ch. nidiphila*, it is a very rare species and, therefore, most likely a Vulnerable species (VU). The rarity of this species is presumably a result of the association of its biology with bats, a group of mammals among which many species are also rare and endangered (Sachanowicz and Ciechanowski 2016). In addition, woodpeckers, whose hollows seem to be the main microhabitat inhabited by the mite species in question, are an endangered group of birds due to the shrinking areas of old-growth forests (Virkkala et al. 1993; Angelstam and Mikusiński 1994; Czeszczewik and Walankiewicz 2006).

## Conclusions

Regarding the decreasing biodiversity of all organisms, including mites (Sullivan and Ozman-Sullivan 2021), any data on the species composition of the acarofauna in a given area are of great value, especially coming from areas of high natural value or those legally protected. Nowadays, however, faunal studies are becoming increasingly rare. This is a substantial loss, as species occurrence data can play the key role in determining the geographical ranges of species, assessing the functioning of ecosystems and the natural value of areas, and, in the long term, also monitoring the changes that occur in the environment. Thus, it is vital that management plans of legally protected areas such as national parks, nature reserves, protected landscapes etc. also take into account microarthropods– both those inhabiting soil, as well as in unstable microhabitats.

The current study has shown that nest boxes, as an artificial type of habitat made by humans, are an important microhabitat for invertebrates, in this case mites from the suborder Uropodina. The presence of boxes in a given area increases the overall biodiversity of Uropodina mites, as evidenced by the obtained MI values, which were higher for the material containing the samples from the examined nest boxes. The study also confirmed that the presence of nest boxes provides a habitat for a rare species of Uropodina, which in the case of this study is *Ch. nidiphila*. Natural habitats of this species are hollows excavated by woodpeckers (Błoszyk et al. 2021), however this was the first time this species was reported in material from bird nest boxes. This species has not previously been reported in nest boxes in other regions of Poland, therefore this evidence expands not only its habitat preferences, but also the previously known area of its occurrence, which now also includes Lower Silesia, Greater Poland, and the Kuyavian region. In the case of nest boxes, however, the species occurs less frequently and in lower number than in woodpecker hollows. However, given the decline in the natural habitat of this species, its occurrence in a microhabitat created by humans, such as nest boxes, can be important for its survival.

## Data Availability

The data presented in this study stored in a computer database called AMUNATCOLL and openly available at: https://amunatcoll.pl/.
